# RISE UP for breast cancer 2024: conference highlights & takeaways

**DOI:** 10.1007/s10549-025-07848-7

**Published:** 2026-01-06

**Authors:** Taylor Glatt, Katherine Leggat-Barr, Nandini Seth, Diane Heditsian, Laura van ’t Veer, Lajos Pusztai, Elissa Price, Nola Hylton, Silvia Formenti, Hope Rugo, Laura Fejerman, Adetunji T. Toriola, Carol Fabian, Andrea De Censi, Daniel Grossman, Olufunmilayo Olopade, Andrea Jackson, Sara Horton, Kim Rhoads, Carol Lange, Virginia Borges, Elizabeth Garner, Judy Garber, Douglas Yee, Laura Esserman

**Affiliations:** 1 Tufts University School of Medicine, Tufts University, Boston, MA USA; 2 University of California, 1825 4Th St, 3Rd Floor, San Francisco, CA 94158 USA; 3 Yale University Cancer Center, Yale University, New Haven, CT USA; 4 Weill Cornell Medical College, New York, NY USA; 5 City of Hope Cancer Center, Duarte, CA USA; 6 UC Davis Comprehensive Cancer Center, University of California, Davis, Davis, CA USA; 7 Division of Public Health Sciences, Department of Surgery, Washington University School of Medicine, Saint Louis, MO USA; 8 Medical Center, University of Kansas, Kansas City, KS USA; 9 Division of Medical Oncology, Department of Medicine, E.O. Ospedali Galliera, Genoa, Italy; 10 Center for Clinical Cancer Genetics and Global Health, The University of Chicago, Chicago, IL USA; 11 Access and Diversity, Quantum Leap Healthcare Collaborative, San Francisco, CA USA; 12 Masonic Cancer Center, University of Minnesota, Minneapolis, MN USA; 13 Division of Medical Oncology, University of Colorado Anschutz Medical Campus, Aurora, CO USA; 14 American Medical Women’s Association, Lexington, KY USA; 15 Dana-Farber Cancer Institute, Boston, MA USA

## Abstract

RISE UP (Revolutionizing Investigations to StEp Up Prevention) for breast cancer brought together leading cancer specialists, women’s health providers, basic and population scientists, regulators, politicians, industry leaders, patient advocates, and more from around the world to discuss and chart a radical rethinking of breast cancer prevention and risk reduction through a lens of hormonal management across a woman’s life course. The presentations at RISE UP were organized to outline a path forward by leveraging what we know about breast cancer biology, early detection, treatment, and endocrine therapy toward a better and sustainable approach for breast cancer prevention. Important conference considerations were to expand our thinking about prevention by broadly considering how the hormonal environment during different life phases or common benign conditions could be better managed to minimize breast cancer risk. This set the stage for transitioning to advances in risk prediction, promising risk-reducing agents, and biomarker-driven trials to test them. Biomarker-based trials discussed focused on 1) lower or intermittent doses of standard prevention agents, 2) drugs already approved for other health purposes, and 3) maximizing benefits from lifestyle interventions alone or in combination. Throughout RISE UP, there was a strong focus on promoting health equity, including comprehensive reproductive health access, equitable representation in clinical trials, and strategies to educate women, providers, and advocates about disparities in care and how to successfully reduce them. The meeting concluded with a competition for innovative approaches to breast cancer prevention that could be integrated into hormonal and women’s health interventions. RISE UP was an innovative conference that provided a forum for cross-cutting topics in women’s health that do not currently exist. The insights shared at RISE UP will be paradigm shifting in breast cancer prevention and women’s health space in the years to come.

## Introduction

RISE UP for breast cancer was designed to fuel innovation and interdisciplinary dialog in women’s health with the goal of rethinking how we can optimize breast cancer prevention. Leveraging what we know about breast cancer biology, screening, treatment, and hormonal management can lead to advances in women’s health by enabling providers from different specialties involved in women’s health to learn from each other. It was held from November 1 to 3, in San Francisco, CA. Over the course of twelve sessions [1], participants learned about tools and strategies for breast cancer classification, imaging biomarkers as early endpoints, efficacious and less toxic therapy, risk prediction, and prevention. We also explored a holistic approach to women’s health, diversity in clinical trials, and examined challenges in contraception research and innovation and breast cancer prevention research through a historical lens. Overall, we investigated how these insights and learnings could be applied to more effective and practical approaches to breast cancer prevention. The meeting ended with an innovative “Shark Tank” competition, where companies pitched ideas for novel prevention agents and strategies with the goal of receiving funding for the best pitch.

RISE UP focused on how we can leverage lessons learned from innovative biomarker-based trials and apply these approaches in the arena of hormonal management with a secondary goal of breast cancer risk reduction. A critical focus was to identify and begin to fill the gaps where knowledge and data are lacking, especially in endocrine management for healthy women. It brought together an interdisciplinary and international group of experts, with the goal of creating new opportunities to collaboratively address the multi-faceted medical issues that many women face before and after breast cancer and charting forward a radical rethinking of how we approach breast cancer prevention.

### Contraceptives: what can they teach us and how could they be utilized in breast cancer prevention?

RISE UP opened with a history of the development of oral contraception (OCP), and how it was initially promoted and adopted, and why research and innovation in this arena has stagnated. OCP’s are a widely used drug [2], with more than 70% of women using these agents across their lifetime [3]. In many cases, OCP’s were initially marketed as a lifestyle drug, with doctors prescribing OCPs both for pregnancy prevention, and to ameliorate other symptoms (acne, Polycystic Ovary Syndrome (PCOS), Premenstrual Dysphoric Disorder (PMDD), painful periods, and more). However, innovation in contraception has been static as the market has become oversaturated with products, missing opportunities to make the pill health promoting in other ways. There has never been an explicit effort to consider how OCPs also reduce the risk for breast cancer. While they reduce the risk of ovarian cancer, this was serendipitous and not planned. OCPs serve as an example of how we can think innovatively, explicitly integrate breast cancer risk reduction, and challenge researchers to think about how we might embark on such an endeavor. These themes were repeated throughout RISE UP.

This history raises the questions: Are there ways we can learn about multiple health-promoting benefits in one trial? Can we increase innovation in products widely taken by women to achieve several health-promoting benefits with one strategy (i.e., contraception, hormonal management, etc.)? These problems require work across disciplines and represent a significant opportunity to improve the health and symptoms of women. Political challenges to this approach abound but are critical to get beyond. Throughout the conference, presenters examined the current body of knowledge, the barriers, and efforts to shift the existing paradigm.

### Overview of treatment trials

The first sessions at RISE UP discussed how the heterogeneity of breast cancer was revealed by biomarker analysis and the subsequent translation of these findings resulted in the development of more individualized approaches which are now used to treat invasive breast cancer. These lessons include how to better classify tumors to tailor treatment approaches, how to quantify treatment effect during pre-surgical systemic therapy (neoadjuvant therapy) with MRI imaging (functional tumor volume change), circulating tumor DNA (timing of resolution or not), pathology (residual tumor burden), and AI to understand less responsive tumors. As a result, therapies are less toxic and more individualized. This is critical to revolutionize treatment and discover new, effective, and less toxic drugs which will be instructive for how to drive progress in prevention.

Trials such as I-SPY 2.2 are utilizing response predictive subtypes to better match treatments to patients and improve outcomes. These trials also highlight new potential drug targets with the goal of improving pathologic complete or near complete responses while also reducing toxicity of therapy by shortening the course of treatment. Further discussed in this session was the application of AI to pathology and the power of this platform to integrate the results of a decade of complex molecular subtyping and simplify it to a read out from standard pathology slides. This would be a lower cost diagnostic that has the potential to improve equity and disseminate this acquired knowledge from the past two decades more broadly across breast cancer care.

### Imaging and promising modifiable imaging biomarkers

The next session focused on how imaging is critical in evaluating response to treatment. The imaging-focused session at RISE UP examined imaging as a predictive marker and early endpoint in clinical trials. Ultrafast Breast MRI is an exciting cutting-edge technology that has the potential to increase both sensitivity and specificity as well as decrease scan time and cost. Multiple scientific abstracts were presented on breast imaging biomarkers. Functional Tumor Volume (FTV) is used in I-SPY 2.2 to de-escalate or escalate therapy based on response, and adjustment based on subtype improves performance. Modifiable biomarkers of breast cancer risk such as mammographic breast density and background parenchymal enhancement (BPE) detected by MRI were presented. Mammographic AI models perform better than BIRADS density in identifying women at risk for breast cancer, especially in the 1–2-year period. How these features could serve as modifiable biomarkers for early endpoints in prevention studies was discussed.

### When less is more in breast cancer treatment

Two debates introduced the topic of how to safely de-escalate therapy. The first focused on early-stage low-risk cancers. As the widespread introduction of mammographic screening and use of MRI (for screening and treatment) increases, a larger proportion of earlier low-risk cancers are being detected. Among post-menopausal women undergoing breast-conserving surgery, in view of the excellent prognosis for most of these cancers, de-escalation of treatment was proposed, specifically lowering the age at which radiation can be avoided and considering when endocrine therapy could be omitted. By characterizing the initial tumor and its response to therapy, the opportunity to avoid radiation all together in some circumstances can be considered. An alternative to omission of radiation was to improve adoption of newer techniques that decrease the number of fractions through accelerated hypofractionation and enable optimal sparing of adjacent organs (heart and lung) by adopting a prone breast radiotherapy technique. In addition, a partial breast radiotherapy approach was discussed as an optimal compromise for most early node-negative breast cancer patients.

The second debate addressed whether systemic therapy could be reduced based on response to therapy, after a complete pathologic response (pCR) is achieved, meaning no invasive tumor is found at the time of surgery in the breast and regional nodes. A lively discussion ensued about the importance of biomarkers and whether they are sufficient to omit further therapy when pCR is reported by pathologists and is used as a surrogate for long-term outcomes. Additional presentations focused on how important it is to either safely de-escalate therapy duration or find ways to ameliorate the many side effects associated with long-term endocrine therapy in women with HR + tumors, specifically those affecting sexual health.

### The roadmap for prevention through leveraging what we know and prioritizing innovation

At RISE UP, a comprehensive roadmap for advancing prevention efforts was established. Driving innovation in prevention requires a strategic framework that builds upon the successes of breast cancer treatment while addressing the distinct challenges inherent to prevention—challenges that treatment-focused trials may not encounter. These challenges were addressed in subsequent sessions and include.

1. Trials of primary prevention are costly, have high safety thresholds, and are hampered by the lack of validated biomarkers to serve as intermediaries for assessing early risk and benefit. Harms may also emerge slowly and irreversibly. Investment in this space is thus inhibited.
2. Pharmacologic prevention requires exposing healthy individuals to products, some of whom will never develop the disease. Therefore, regulators require low-risk profile drugs, making this a particularly undesirable area for industry investment.
3. Long-term endocrine interventions may have undefined and unanticipated systematic effects, complicating risk assessment. Women’s reproductive health is politically charged**,** further deterring investment.
4. For years, women’s health has suffered from underinvestment, meaning drugs that specifically work in the female hormonal environment are more challenging to develop.

### Utilizing modifiable biomarkers to predict risk and act as trial endpoints

Researchers at RISE UP presented progress in developing critically important biomarkers to advance risk prediction and risk reduction to spur progress in prevention. Despite their importance to prevention efforts, modifiable and scalable biomarkers for identifying risk and risk reduction remain elusive. Prevention interventions are most useful when those at high risk for developing breast cancer can be identified and would be particularly important if they could predict the type of breast cancer most likely to develop. This session focused on how we identify targetable pathways for prevention in women, creating specific biomarkers that can estimate who is at risk for what type of cancer. Currently, traditional risk factor-based models are getting better, but still have room for improvement, where, for example, even the best models, like polygenic risk, still have poor to fair discriminatory ability. This means there is a huge opportunity to enhance these models to improve their discriminatory capacity. There is much ongoing research on utilizing and classifying omics of common pathways that increase breast cancer risk (i.e., linking early life adiposity, dense breast tissue) to estimate who is most at risk for breast cancer. Similarly, several presenters focused on opportunities to accelerate learning using these risk tools as modifiable markers for trials. An exciting example was the use of imaging biomarkers, including how AI interpretation of mammographic images could identify women at risk and who would benefit most from additional contrast-based imaging and risk reduction. AI has the potential to transform how we solve classification of breast density and identify 10% of women with dense breast tissue as high risk, rather than 50% (BIRADS C and D) of the population of women aged 45 and above [4]. There is also strong potential for combining polygenic risk with AI imaging and EHR data for greater risk discrimination. Further development of tools that identify *who* is at risk for breast cancer will not only revolutionize personalized prevention, but these surrogates can improve our ability to invest in trial endpoints for prevention interventions, including trials measuring prevention as a secondary endpoint.

### Utilizing modifiable endpoints and spurring innovation in prevention products

It is not enough to solely predict who is at risk for breast cancer. Even with this additional knowledge, we need tangible interventions to reduce breast cancer risk that ideally are subtype specific. This requires developing new and improving existing effective drugs to minimize side effects, along with better methods to predict how well these interventions will reduce breast cancer risk.

There is poor uptake of standard primary prevention interventions (especially the selective estrogen receptor modulators tamoxifen and raloxifene), particularly in women with no prior diagnosis of ductal carcinoma in situ (DCIS), lobular carcinoma in situ (LCIS), or atypical ductal hyperplasia (ADH). This is due to a confluence of factors, such as 1) side effects of intervention, 2) incomplete efficacy, 3) difficulty in predicting short-term risk of breast cancer, and 4) determining response to the intervention on an individual level, made more difficult because prevention biomarkers are still in development. For prevention medications to be efficacious, tolerable, and have broad uptake, satisfying each criterion is critical.

At RISE UP, promising directions for interventions were discussed that satisfy the criteria to make these drugs more tolerable and increase uptake. For one, designing interventions to address a current problem or symptom that a woman is struggling with, in addition to breast cancer risk represents a particular promise. Examples include diet and exercise interventions in women with obesity, GLP-1 and dual GLP-1/GIP receptor agonists (tirzepatide) in women with obesity, metformin in women with insulin resistance, and bazedoxifene and conjugated estrogens in peri- and postmenopausal women with vasomotor symptoms. By utilizing a drug that has symptom amelioration in addition to breast cancer risk reduction, it is likely to be more tolerable and more desirable to women who are taking it.

Secondly, lower doses of standard prevention drugs could reduce side effects, including low-dose tamoxifen and low-dose exemestane. Evidence suggests that lower doses of these medications are as efficacious (defined by recurrence of breast cancer) and cause significantly lower side effect burden, which could also increase uptake by women [5]. This is particularly important for those at risk for endocrine sensitive tumors. Similarly, one can combine interventions to improve efficacy without significantly increasing side effects such as low-dose tamoxifen with diet and exercise or high-dose omega-3 fatty acids. This could provide broader risk protection for both endocrine sensitive and insensitive tumors, of particular importance as we need interventions that treat individuals who are at risk for all types of cancer.

While our current interventions can potentially be tailored to improve efficacy while reducing toxicity, it is also important to continue to innovate new endocrine agents with potentially improved side effect profiles and/or efficacy. Promising examples include acolbifene and lasofoxifene. Funding new research that explores agents with lower toxicities is vital to increasing uptake of these adjuvant therapies and reducing recurrence.

### Hormonal environment milieu & opportunities for prevention

Understanding the hormonal environment is critical to optimizing gains in prevention. Across a woman’s lifetime hormonal levels constantly change within new contexts. Complex steroid family receptor-driven networks are likely as complex as the immune system and are currently being studied. Importantly, although they are distinct receptors, estrogen receptor-alpha (ER) and progesterone receptor isoforms (PRs) act either antagonistically or collaboratively, driving proliferation but contributing to cancer stem cell expansion and therapy resistance. This improved understanding will help us target and increase efficacy of hormonal medications that are already in use and those in development. These receptor interactions can provide greater context to tumor biology, especially in recurrent and metastatic disease, and contribute to the development of targeted treatment and prevention.

Similarly, since the Women’s Health Initiative (WHI) was published 20 years ago, we have learned more about the impact of various hormones on breast cancer risk. For example, progesterone comes in many forms and these agents are not equivalent. Several landmark studies have identified various progestins that have contributed to breast cancer risk. For example, MPA (medroxyprogesterone acetate) is the most widely used progestin (a synthetic form of progesterone) and has been linked to breast cancer risk in most trials examining hormone replacement therapy. MPA is shown to increase cell proliferation, and androgenic activity significantly more than natural progesterone. However, other progesterone’s, such as drospirenone (DRSP), have a much lower impact on cell proliferation or androgenic activity. We must not think monolithically about hormones but rather approach them with nuance. Unfortunately, none of these new agents have been studied widely since the WHI. There is a clear need to initiate new large-scale studies of agents in current use in order to inform women about risk and benefit.

### A real-life example of how prevention could be incorporated into existing products

A real-world example of these principles in action is the development of contraceptives that not only provide reliable pregnancy prevention but also promote overall health and reduce breast cancer risk through innovative design.

Ulipristal acetate (UPA), a selective progesterone receptor modulator currently approved as emergency contraception, has potential protective effects against breast cancer and was studied to treat fibroids. This compound represents a unique opportunity to develop products that improve quality of life and symptoms related to fibroids while also reducing risk for breast cancer. In a post marketing study, rare risks of liver toxicity (1 in 100,000 women) were observed which halted further development of UPA. However, the threat that fibroids pose to women is underappreciated, particularly in black women. This medication, that is clearly effective in reducing fibroid size and hysterectomy rates due to fibroids, has the potential to be risk reducing for breast cancer because it is an anti-progestin. This underscores the potential opportunity to design medications that address prominent women’s health issues while also reducing breast cancer risk.

### The impetus for trial inclusiveness and advocating for reproductive autonomy

A central theme of RISE UP was how we can better understand, rethink, and address disparities in women’s health, specifically in the areas of abortion care, maternal mortality, and breast cancer. The Dobbs decision resulting in the restriction of abortion access in many states disproportionately impacts those already marginalized. Women in states with abortion bans must travel across state lines or use telehealth to obtain care, providers are forced to consider their license rather than solely focusing on the patient, and care is sometimes delayed or denied. When a community loses an abortion provider, they also lose access to other reproductive healthcare services, lowering the quality of care accessible to many patients. In the ongoing qualitative Care Post-Roe study from UCSF’s Advancing New Standards in Reproductive Health (ANSIRH) [6], a medical student interviewee described a particularly harrowing experience of a patient who was forced to keep her pregnancy and then endure labor even though her fetus was not viable and would die in utero or immediately after birth. Instead of having access to an abortion, the student watched the patient give birth and hold her stillborn child, highlighting the trauma patients, students, and providers must face in the wake of the Dobbs decision. Dobbs also changed the landscape of medical education and training. Many states with OB/GYN residency programs now must coordinate out-of-state rotations to teach their residents comprehensive women’s reproductive care, and trainee applications in banned states have plummeted.

### A deeper dive into disparity

In addition to the inequitable effects of the Dobbs decision, significant disparities persist in maternal and breast cancer mortality between Black women and White women in the USA. Black women face a 40% higher mortality rate following a breast cancer diagnosis, with increased mortality observed in both HR-positive and HR-negative tumors. Given the heterogeneity of breast tumor biology, it is essential to enroll Black women by self-report in clinical trials in ways that reflect their specific burden of disease. Doing so is critical to advancing targeted therapies for those who face disproportionately high mortality rates.

Black women also experience worse outcomes in common gynecologic conditions, such as fibroids, infertility, and pelvic floor disorders. One emerging biological pathway that may underlie these inequities is the cumulative impact of chronic stress—particularly stress induced by structural racism—which has been shown to drive persistent low-grade inflammation. This heightened inflammatory state can contribute to tumor progression in breast cancer as well as the development and severity of fibroids, impaired reproductive function, and weakened pelvic support structures. Importantly, besides chronic stress and inflammation, significant barriers in access to care and systemic bias exacerbate disparities in Black women’s health outcomes. These effects highlight the urgent need for intervention on multiple levels.

We must not only invest in treating these conditions—we must also commit to preventing the disparities that shape outcomes in both breast cancer and gynecologic health through collaborative, innovative, and equity-focused solutions.

### Postpartum-associated breast cancer and associated disparities

With increasing incidence and higher rates of metastases and death in young women, pregnancy-associated breast cancer is an emerging opportunity to address disparities and implement prevention strategies. There are potential biomarkers such as SEMA7A and pathology features that appear to be associated with pregnancy-associated breast cancer and recurrence. Importantly, during the period of weaning, involution occurs and is thought to be a window of susceptibility for cancer initiation. There are simple interventions, including short-term use of non-steroidal anti-inflammatory (NSAIDS) agents that could potentially abrogate risk. This could be a simple and significant opportunity for breast cancer risk reduction and should be a priority for study.

Presentations in this session drew attention to breastfeeding to reduce breast cancer risk and lower incidence of triple-negative breast cancer (TNBC) in Black communities. Breastfeeding duration and younger age at first birth are known risk factors for TNBC and vary by race [7]. Investigators estimate that up to 15% of annual new TNBC in Black women and 12% in White women might be avoided by supporting breastfeeding for longer than 6 months [8]. This emphasizes the need to address social barriers black mothers face, such as decreased job security and maternity leave, that prevent them for breastfeeding for long periods of time. Further, a global disparity exists in access to detection and treatment with differences in maternal mortality rates and mortality from breast cancer in women under age 50, emphasizing a need to study parity, weaning, and breast involution as a risk factor of breast cancer. Since pregnancy-associated breast cancer is poorly understood, we miss the opportunity to learn how it can lead to metastasis and how the cessation of breast feeding can serve as a key prevention window. Insights into when and how to intervene in the weaning process provide a promising future to reduce the chance that these cancers will develop.

### The importance of policymakers and politicians in addressing disparities in clinical trials

To start addressing disparities, all women, especially women of color, must be offered the same opportunities for clinical trials. One session specifically explored financial and systemic challenges that prevent historically underserved populations from participating in clinical trials and highlighted successful strategies to address them. A case study of the I-SPY2 Trial, featuring site leadership and research staff from USC/LA County, shared best practices for recruiting and retaining diverse participants in a safety-net setting. A former I-SPY2 participant and advocate provided a powerful patient perspective, emphasizing the importance of trust, navigation, and culturally responsive engagement when she shared, she was only offered clinical trial opportunities at her second opinion appointment. The session then discussed policies pertaining to socioeconomic status and the ability to pay and seek care at institutions offering clinical trials. Longstanding legislation in California, SB37 (2002), mandates that if someone is eligible for a trial, they can enroll in the trial and their insurer must cover the standard of care costs (up to 120% of Medicare costs). However, many are not aware of this law. Some states have developed similar laws though many gaps remain. National legislation, HR8546, Clinical Trial Coverage Act of 2022, has been proposed to cover people everywhere, to make this a reality for everyone. It has yet to pass, but it is being actively advanced. Patients deserve to be informed of all possible treatment options without consideration of their racial or economic background. The founders of the Lazarex Cancer Foundation and Tigerlily Foundation also described how non-profit support and patient advocacy efforts can close gaps in access, improving awareness of breast cancer clinical trials. Together, this session highlighted how equity in access to trials requires aligning policy, infrastructure, and patient-centered solutions.

RISE UP both highlighted the persistent disparities faced by women and illuminated ways we may be able to reduce these disparities with inclusive clinical trials, legislation, and innovation in women’s health.

### A grand finale: increasing investment and innovation in women’s health

The final RISE UP session brought together an interdisciplinary panel of judges representing the scientific/regulatory, industry, venture capital, and consumer/advocacy sectors for a Shark Tank-like session. Four pitches were presented and evaluated by a panel of experts. Three proposals focused on repurposing existing medications—mifepristone, tirzepatide, and low-dose testosterone combined with an aromatase inhibitor (T + Ai)—as breast cancer risk-reducing agents, aligning with RISE UP’s mission to drive innovation in prevention strategies. The fourth pitch focused on personalized exercise regimens for those in treatment and how to incorporate exercise as a prevention tool. Due to the strengths of the proposals, the reviewers funded all the finalists and they will present their progress at the second annual RISE UP, to be held February 19–21, 2026.

These insights underscore the need for cross-disciplinary collaboration to advance safe, tolerable, and effective risk-reducing strategies for individuals at high risk of breast cancer. It also represented excitement and opportunity to engage investment and industry leaders to form partnerships to better succeed in these goals. This collaboration is critical to achieving a women’s health revolution.

## Conclusion

To revolutionize our approach to women’s health—and meet the challenge of finding innovative ways to optimize prevention across a woman’s lifespan—we must integrate management strategies for her symptoms and needs throughout her lifetime, promoting equitable, accessible care for all. To succeed, we must engage all disciplines, including breast cancer providers and researchers, primary care clinicians, pathologists, radiologists, OB/GYNs, patient advocates, policymakers, regulators, venture capitalists, and industry leaders.

RISE UP provided a critical forum that sparked innovation, discussion, and collaboration to develop meaningful solutions. The speakers and competition judges reflected on the role of biomarkers as reliable intermediate endpoints to accelerate progress in prevention, the importance of minimizing side effect profiles for medication tolerability, the opportunity to integrate prevention as a secondary outcome in drugs that address other important health needs and rethinking the design of clinical trials for such approaches. It has been nearly 20 years since the last major women’s health initiative. With all the new knowledge we have generated and with the consistent threats to women’s health autonomy, the urgency for a new, cross-disciplinary collaboration has never been greater. The time to act is now.

2024 Abstracts: 2024 RISE UP for breast cancer abstract collection. Breast Cancer Res Treat 211 (Suppl 1), 1–42 (2025). 10.1007/s10549-025-07692-9

2024 Agenda: https://riseup.ucsf.edu/2024-rise-conference-presentations

## Data Availability

No datasets were generated or analysed during the current study.

## References

[1] “2024 RISE UP for breast cancer abstract collection,” *Breast Cancer Res. Treat.*, vol. 211, no. 1, pp. 1–42, Jun. 2025, 10.1007/s10549-025-07692-9. 10.1007/s10549-025-07692-9 40327265

[2] Hall KS, Trussell J (2012) Types of combined oral contraceptives used by U.S. women. Contraception 86(6):659–665. 10.1016/j.contraception.2012.05.017 22770787 10.1016/j.contraception.2012.05.017 PMC3469779

[3] B. Frederiksen, U. Ranji, A. Salganicoff, and M. L. Published, “Women’s Sexual and Reproductive Health Services: Key Findings from the 2020 KFF Women’s Health Survey,” KFF. Accessed: May 09, 2025. [Online]. Available: https://www.kff.org/womens-health-policy/issue-brief/womens-sexual-and-reproductive-health-services-key-findings-from-the-2020-kff-womens-health-survey/

[4] Sprague BL et al (2014) Prevalence of mammographically dense breasts in the United States. J Natl Cancer Inst 106(10):dju255. 10.1093/jnci/dju255 25217577 10.1093/jnci/dju255 PMC4200066

[5] Lazzeroni M et al (2023) Randomized placebo controlled trial of low-dose tamoxifen to prevent recurrence in breast noninvasive neoplasia: a 10-year follow-up of TAM-01 study. J Clin Oncol 41(17):3116–3121. 10.1200/JCO.22.02900 36917758 10.1200/JCO.22.02900

[6] “Advancing New Standards in Reproductive Health,” ANSIRH. Accessed: Sep. 30, 2025. [Online]. Available: https://www.ansirh.org/home

[7] Shinde SS, Forman MR, Kuerer HM, Yan K, Peintinger F, Hunt KK, Hortobagyi GN, Pusztai L, Symmans WF (2010) Higher parity and shorter breastfeeding duration: association with triple-negative phenotype of breast cancer. Cancer 116(21):4933–4943. 10.1002/cncr.25443 20665494 10.1002/cncr.25443

[8] Chehayeb RJ, Odzer N, Albany RA, Ferrucci L, Sarpong D, Perez-Escamilla R, Lewis JB, Phipps AI, Meisner A, Pusztai L (2025) Breastfeeding attributable fraction of triple negative breast cancer in the US. NPJ Breast Cancer 11(1):40. 10.1038/s41523-025-00755-6 40328734 10.1038/s41523-025-00755-6 PMC12055980

